# Neuralgia

**Published:** 1894-01

**Authors:** 


NEURALGIA.—Neuralgia, or neural hyperaesthesia, is the generic name for a number of diseases of which the chief symptom is pain following the course of a nerve or group of nerves and generally fixing itself where the nerve emerges from a foramen or at a superficial point just before it dips into a fascia. This designation is indefinite and leaves room for many central lesions to be classed as neuralgia, which have an entirely different pathology, and consequently require different treatment. No pain that is due to a disease of the brain or spinal cord should be called neuralgia, but that alone which is limited to a nerve in some part of its course. It is most commonly located in one of the branches of the fifth pair, oftener in the opthalmic division, when we have that condition generally known as hemicrania. This form is usually intermittent or periodic, coming on in the morning and subsiding at nightfall. The stomach is often nauseated and this reflex manifestation, especially in females, gives it the name of "sick-headache," which is one of the most troublesome affections the physician has to encounter. When that part of the face from the lower eyelid to the mouth, including the side of the nose, cheek and upper jaw is involved, we have the lesion seated in the superior maxillary division. When the lower jaw is involved, the disease is in the inferior maxillary division. When the neck and back part of head are the seat of pain, the first four cervical nerves are involved. This form is not so acute or painful as that of the fifth year.

When the neck and shoulder are the pathological points, the disease is seated in the brachial plexus of the lower five cervical and first dorsal nerves. This variety generally follows exposure to cold, traumatism or a rheumatic diathesis. The intercostal nerves may also be affected, when we have intercostal neuralgia or pleurodynia. If the pain is in the back, we have involvement of the dorsal nerves, this is sometimes thought to be muscular rheumatism. If we have the lumbar plexus as the seat of pain, what is termed "lumbago" is the result. The sciatic nerve may be affected, when we have either a neuritis or a functional neurosis. It is called "sciatica" and generally is accompanied by a history of rheumatism or gout, and is often a very stubborn disease. Coceydynia or neuralgia of the coccyx and pododvnia neuralgia of the heel, are also varieties of this disease that often occasion the physician endless trouble. Before we undertake the treatment of this disease, it is vitally important to know as much as we can of its etiology and complications. The success of our treatment depends largely upon our knowledge of the morbid process in operation and neuro-pathological conditions transmitted by the parent to the offspring, as well as the quality of the blood and nutrition of the body. With an inherited tendency to neuralgia, followed by anemia, depressed vitality of the system, and exposure, especially to malarial influences, it is very easy to establish the disease on an enduring basis, but by removing or counteracting these factors, followed by proper medical treatment the patient may, generally, be restored to health. If the cause is malarial or syphilitic and the disease is of an intermittent type, it yields more readily to treatment, but when these causes do not operate and the patient is advanced in years, the prognosis is not so favorable. Often in neuro-pathic families, when there is an enfeebled nervous system, the least exciting cause will develop neuralgia and to prevent such results, the nutritive processes must be supported until the nerves, blood and every organ are restored to the healthiest, normal condition, very often when this is done, specific treatment is not necessary, as the pain vanishes as suddenly as it appeared.—*St. Louis Medical Era.*

